# The impact of obesity on neuropsychological functioning in adults with and without major depressive disorder

**DOI:** 10.1371/journal.pone.0176898

**Published:** 2017-05-05

**Authors:** Maria R. Restivo, Margaret C. McKinnon, Benicio N. Frey, Geoffrey B. Hall, Wasimuddin Syed, Valerie H. Taylor

**Affiliations:** 1 Womens College Research Institute, Women’s College Hospital, Toronto, Ontario, Canada; 2 Department of Psychiatry and Behavioural Neurosciences, McMaster University, Hamilton, Ontario, Canada; 3 Mood Disorders Program and Women’s Health Concerns Clinic, St. Joseph’s Healthcare Hamilton, Hamilton, Ontario, Canada; 4 Department of Psychology, Neuroscience & Behaviour, McMaster University, Hamilton, Ontario, Canada; 5 Faculty of Health Sciences, McMaster University, Hamilton, Ontario, Canada; 6 Department of Psychiatry, University of Toronto, Toronto, Ontario, Canada; University of North Carolina at Chapel Hill, UNITED STATES

## Abstract

**Background:**

Evidence suggests obesity exerts a negative impact on cognition. Major Depressive Disorder (MDD) is also linked to problems in cognitive functioning. Obesity is highly prevalent in individuals with MDD and is linked to a failure to return to a full level of functioning. The study’s objective was to investigate the effect of obesity on cognitive impairment in participants with MDD.

**Methods:**

This study compared cognitive performance in obese individuals with MDD and two control populations (obese individuals without a psychiatric illness and non-obese controls). A standardized battery of neuropsychological tests specifically designed to assess performance in declarative memory, executive functioning, processing speed and attention was administered. Mood ratings, physical measurements, nutritional and health questionnaires were also completed.

**Results:**

We observed a consistent pattern across measures of memory, executive functioning, attention and processing speed. Whereas healthy controls performed better than both bariatric groups across the majority of measures administered, bariatric controls tended to outperform bariatric MDD patients.

**Limitations:**

The overall sample size of our study was small and thus largely explorative in nature. However, it provides compelling results (while controlling for extraneous variables such as medication load, nutritional status and common metabolic comordidities) that strongly urges for further investigation and study replication with larger sample sizes.

**Conclusions:**

We found obesity has a subtle impact on cognition in obese individuals, and when obesity is present in individuals with MDD, this impact may be significant. It is important to minimize all modifiable variables that can add to cognitive burden in this population.

## Introduction

Obesity is associated etiologically with cardiovascular disease, in part due to its contribution to risk factors such as dyslipidemia, hypertension and type II diabetes (T2D) [1]. As a consequence, public health interest in obesity prevention and treatment has been significant [2]. This effort has begun to target specific populations, as there is an inherent increased vulnerability towards weight gain associated with mental illness. Individuals with major depressive disorder (MDD), an illness predicted to be the main cause of disability worldwide by 2020 [3], have a 21% higher risk of developing obesity than the general population according to the National Comorbidity Survey-Replication (NCS-R) [4]. The deleterious effects of obesity on peripheral systems have been well elucidated but its impact on central brain function remains much less well understood [5]. An emerging area of investigation suggests that adiposity may negatively impact cognitive functioning [6–8]. For example, studies of obese adults seeking bariatric surgery have shown significant impairment on measures of executive functioning in particular prior to surgical intervention [6, 9]. Although executive functioning (higher-order cognitive processing) is the cognitive domain most often affected, performance on tasks of memory also point towards a potential negative association with obesity [10].

The impact of obesity among individuals with mental health conditions and, in particular, MDD, may be particularly problematic. Here, individuals with depression show impairment relative to matched healthy controls across multiple cognitive domains, including memory, processing speed, and cognitive flexibility [11–16]. Additional studies point towards alterations in performance on executive functioning tasks involving the selection, timing, monitoring and interpretation of behavior, and on measures of working memory and selective attention [11, 17, 18].

Despite clear links between obesity, and cognitive functioning and MDD, the extent to which obesity impacts cognitive functioning in individuals with MDD is absent from the literature. Even with the greater prevalence of overweight or obese individuals in the MDD population [19–21], participant weight is not routinely reported as a demographic characteristic or potential covariate in reviews on cognition in this population [11, 15, 22–26]. Given that impaired cognition is often linked with a failure to return to full functioning, despite a remission in other symptoms [12, 27, 28] and that obesity is a potentially preventable or modifiable risk factor, there is an urgent need to explore this association.

We aimed to examine the impact of obesity and MDD on cognitive function in an adult population (age 18–60). Cognitive performance was assessed in obese individuals seeking bariatric surgery, with and without MDD, and compared to healthy control (non-depressed, normal body mass index [BMI)]) individuals. Here, we hypothesized that healthy controls would outperform both bariatric (obese) participant groups on measures of cognitive performance. We also postulated that depression would have an additive effect, wherein bariatric participants with MDD would perform more poorly than bariatric controls (free of psychiatric illness). In addition, we examined how important potential confounding variables not routinely investigated in previous studies, such as nutritional intake and the presence of medical illnesses commonly co-morbid with obesity, might impact these associations.

## Methods

### Participants

This study was conducted at St. Joseph’s Healthcare Hamilton and received full ethics approval from the Hamilton Integrated Research Ethics Boards (09–3254). Approximately 3100 charts from the bariatric surgery program were reviewed for potential study eligibility and 683 participants were contacted. Healthy controls were recruited from the community. Full recruitment procedures and a full description of study protocol are outlined in Restivo et al. [29]. *Inclusion Criteria* for all groups was as follows: age 18–60 years, ability to provide informed consent, and native English speaker (or having learned English by age 6). Additionally, healthy controls were required to have a BMI between 18.5–24.9 (normal range) [30]. *Exclusion Criteria* included the presence of a current or pre-existing neurological condition (e.g., epilepsy, severe head trauma) or unstable and/or severe medical condition (e.g., cancer, severe heart attacks), having been administered any of the cognitive study measures within the past 12 months, a history of a confirmed learning disorder or developmental disability diagnosis (e.g., attention deficit hyperactivity disorder) or a Full Scale Intelligence Quotient (FSIQ) < 70, an inability to complete the testing (e.g. due to a hearing or vision impediment), and the presence of alcohol or substance abuse within the last 6 months or lifetime dependency. In addition, presence of a past or current psychiatric condition was exclusionary for both the healthy BMI and bariatric (obese) non-psychiatric control groups, while having been administered electro-convulsive therapy (ECT) within the last 24 months is an exclusion criterion for MDD bariatric patients.

Of those who met criteria and chose to participate, 82 provided informed written consent and were enrolled, and 78 completed the study. Distance from testing site (many bariatric clinic patients lived out of town), schedule conflicts/inflexibility, and unwillingness to undergo an MRI testing session were the most commonly cited reasons for participation decline. Two participants (of the 78 enrolled) chose to no longer pursue surgery and did not complete their scheduled neuropsychological testing study visits as a result and an additional two participants withdrew consent due to scheduling conflicts. Of the 78 who completed neuropsychological testing, two participants were excluded from analysis, one for disclosure of exclusionary medical comorbities during a study visit and a FSIQ <70 and the second for a diagnosis of previous substance use dependency. Here, we report on the three groups for which enrollment and data collection has been completed (healthy controls, bariatric controls and bariatric MDDs); a total of 66 participants are included in this sample. An additional 10 participant representing bariatric patients with Bipolar Disorder are not reported in this study (recruitment for bariatric patients with Bipolar Disorder is currently ongoing). In order to investigate the representativeness of the bariatric study participants in relation to the wider bariatric surgery patient population, descriptive summary information was obtained from the Ontario Bariatric Registry (ORB), a province-wide (multi-site) database that collects medical and demographic data on patients seeking bariatric surgery in Ontario as of 2010 [31]. A comparison of demographic and clinical characteristics of the study sample with data available from the provincial registry is available in Table 1.

**Table 1:** Comparison demographic characteristics of study sample, St. Joseph’s Healthcare Hamilton bariatric surgery program candidates and provincial bariatric surgery candidates (as reported by Anvari, M., Sharma, A., Yusuf, S., et al., 2015).

	Study Sample	SJHH	All Ontario Centres
**Age (Mean)**	43.7 (10.7)	46*	45*
**Male:Female (%)**	9.1:90.9	20.0:80.0	19.5:81.5
**Type II Diabetes (%)**	29.5	33.3	32.7
**Hypertension (%)**	39.5	46.0	47.4
**Hyperlipidemia (%)**	30.2	36.5	33.7
**BMI (Mean)**	44.2 (3.8)	49.4	48.6

*Standard Deviation information was not available from the Bariatric Registry

### Independent variables

BMI groups were defined based on the criteria established by the National Heart Lung, and Blood Institute, Obesity Education Initiative: normal weight (18.5–25.0 kg/m^2^) and obese (≥30 kg/m^2^) [32]. BMI for the bariatric participants ranged from 37.0 (class II obesity) to 55.7 (class III or morbid obesity), with 83.0% of bariatric participants falling in the class III range. Psychiatric status (current and lifetime) was evaluated via the Structured Clinician Interview for DSM-IV–Axis I (SCID-I) [33].

### Covariates

An extensive list of corollary information was also obtained. Data from administered standardized questionnaires, clinical interviews, and participant charts and medication profiles were collected in order to identify and control for potential confounders.

### Medical

Anthropomorphic and medical comorbidity data are shown in Table 2. The weight, height, BMI, waist and hip circumferences, heart rate, average systolic and diastolic blood pressures (averaged from a left and right arm independent reading), and a random glucose ‘finger prick test’ value (taken a minimum 2 hours after having last eaten) was obtained for all 3 groups. In addition, lipid profile values and hemoglobin (Hb) A1c values were obtained for bariatric patient groups. Presence or absence of dyslipidemia was self-reported by healthy controls (individual bloodwork was not made available for this group); no healthy controls reported current presence of dyslipidemia. The Berlin Sleep Questionnaire [34], which assesses the risk level for current Obstructive Sleep Apnea (OSA) or sleep disordered breathing, was also completed by each participant. Participants were then coded as high-risk or low-risk as per Berlin Sleep Questionnaire staging, with participants previously diagnosed with OSA that was currently treated and controlled by a Continuous Positive Airway Pressure (CPAP) ventilator coded as low-risk. Nutritional intake was assessed via a non-consecutive 3-day dietary record (Food Frequency Questionnaire [FFQ]), with one day being a weekend day [35]. In addition to obtaining information on overall average daily caloric intake, diet component analysis was also completed by examining total daily caloric intake, cholesterol, fibre, sugar, sodium and percent and total intake of proteins, carbohydrates, and fat. Smoking was coded as a dichotomous variable (current versus non-smoker). Participants were asked to provide a complete listing of current medications, vitamins, and herbal supplements (including dosage and indication) during their first study visit; medication history was also confirmed via data extraction of bariatric patients’ medical charts and recorded clinic staff encounters. Following previously employed methodology by Sackeim (2001) [36] and Hassel et al. (2008) [37], we generated a composite measure of total (psychotropic) medication load based on dosage and medication class for each bariatric MDD participant.

**Table 2:** Demographic, clinical and medical characteristics of study sample.

	Healthy Controls (n = 20)	Bariatric Controls (n = 25)	b-MDD (n = 21)
**Age (Mean, SD)**	43.8 (11.0)	43.9 (10.7)	43.2 (10.9)
**Sex (Male:Female)**	2:18	2:23	2:19
**Years of Education**◊	16.1 (2.3)	14.2 (2.1)	14.2 (2.3)
**Ethnicity (Caucasian %)**	85	95.2	85.7
**BMI**◊	22.4 (2.0)	44.7 (2.9)	43.7 (4.8)
**Weight (kg)** ◊	60.3 (7.1)	122.0 (10.5)	116.3 (14.5)
**Height (cm)**	164.0 (8.3)	165.2 (4.3)	163.0 (6.6)
**Waist Circumference (cm)** ◊	74.6 (5.2)	123.5 (10.2)	124.1 (12.0)
**Hip Circumference (cm)** ◊	97.7 (5.8)	139.7 (8.0)	134.4 (11.8)
Hypertension* **(%)**◊	0.0	43.5	35.0
Average Systolic BP** **(mmHg)** ◊	119.8 (9.3)	134.3 (18.0)	132.5 (10.5)
Average Diastolic BP** **(mmHg)**	74.6 (16.2)	77.6 (7.0)	76.5 (11.2)
Average Heart rate**	73.4 (12.8)	82.5 (13.1)	80.1 (10.7)
T2D(%)***◊	0.0	33.3	25.0
**HbA1c** ◊	n/a	0.059 (0.019)	0.059 (0.005)
**Random Glucose Test**	5.8 (1.6)	6.1 (2.4)	5.2 (0.8)
Hyperlipidemia****◊	0.0	24.0	33.3
**Total Cholesterol**	n/a	4.60 (0.94)	4.74 (0.95)
**HDL**	n/a	1.26 (0.33)	1.15 (0.27)
**LDL**	n/a	2.65 (0.67)	2.95 (0.87)
**Triglycerides**	n/a	1.53 (0.88)	1.56 (0.58)
**OSA Risk (%High Risk)** ◊	0.0	50.0	33.0
**HAM-D**◊	1.6 (2.9)	1.5 (1.8)	6.6 (4.3)
**BDI**◊	1.9 (5.8)	8.3 (8.6)	17.5 (10.0)
**YMRS**◊	0.6 (0.9)	0.5 (0.7)	2.6 (2.5)
**ASRM**	1.6 (2.6)	3.1 (3.0)	2.7 (2.7)
**SDS (Averaged Across Domains)** ◊	0.0 (0.0)	2.7 (2.7)	4.7 (4.8)
**CFQ Total**◊	22.9 (10.6)	25.2 (6.4)	40.5 (18.6)

*Borderline hypertension was collapsed into the hypertension group (Borderline was defined as Systolic BP between 130 and 139)

**Two independent measures, 1 minute apart were obtained

***Borderline, well-controlled, and sub-optimally controlled TDII status were collapsed

****Elevated lipid value status was also included as hyperlipidemia

^◊^Signficant group effect found (p <0.05)

ABBREVIATIONS: ASRM = Altman Self-Rating Mania Scale; BDI = Beck Depression Inventory; BP = Blood Pressure; CFQ = Cognitive Failure Questionnaire; Ha1bc = Haemoglobin A1c; HDL = High-density lipoprotein; LDL = Low-density lipoprotein; SDS = Sheehan Disability Scale; T2D = Type 2 Diabetes; YMRS = Young Mania Rating Scale

### Demographic

Age at time of neuropsychological testing, years of education, sex, marital status and ethnicity was collected for each participant (see Table 2). The Cognitive Failure Questionnaire (CFQ) [38] was used to assay subjective sense of cognitive dysfunction, while the Sheehan Disability Scale (SDS) was used to quantify functional impairment across 3 life domains (Work/School, Social Life, and Family Life/Home Responsibilities) [39].

### Psychiatric

Mood rating questionnaires were administered on the day of testing or within 2 weeks of the study visit. The Beck Depression Inventory (BDI) [40] and Hamilton Rating Scale for Depression-17 (HAMD-17) [41] were used to examine depressive symptoms while the Altman Rating Scale for Mania (ARSM) [42] and Young Mania Rating Scale (YMRS) [43] were used to control for mania symptoms. Additionally, the Childhood Trauma Questionnaire was administered to control for previous trauma exposure [44] (a potential confounder in MDD populations). Current and past psychiatric morbidities were captured through the SCID-I, and additional information regarding MDD illness burden including illness age of onset and number of episodes was also obtained (clinical characteristics listed in Table 2).

### Neuropsychological testing

A standardized battery of neuropsychological tests aimed at establishing pre-surgical (baseline) performance on tests of declarative memory, executive functioning, processing speed and attention was administered. These cognitive domains were chosen after our review of the literature indicated these cognitive domains as being most susceptible to impairment in metabolically dysregulated populations [45, 46]. The following battery of tests was administered (see Table 3 for further test measure details):

*Declarative memory function*: California Verbal Learning Test II (CVLT) [47], Wechsler Memory Scale III—Logical Memory subtest (WMS-III) [48] and Brief Visuospatial Memory Test–Revised (BVMT-R) [49].
*Executive functioning and attention*: Controlled Oral Word Association Task (COWAT) [50], Stroop Colour and Word Test Sensitivity to Interference (Golden version), (51), Wisconsin Card Sorting Task (64-item version) (WCST) [52], Colour Trails Test (CTT) Part B, [53] and Paced Auditory Serial Attention Test (Victoria Computerized Adaptation) (PASAT) [54].
*Processing Speed*: Colour Trails Test A [53], Stroop Colour and Word Test (Golden version): Word reading and colour identification subtests [51].

**Table 3:** Test battery description—Cognitive measures and variables investigated.

Battery (Domain)	Test Measure	Test Variable	Measure and Purpose
**Executive Function**	BVMT-R	Learning Index	Rate/amount of visuospatial learning over successive stimulus presentation trials
	COWAT	Total Responses–FAS Letter (Phonemic Fluency)	Verbal association fluency (spontaneous word production)
	COWAT	Total Responses–Animal (Categorical Fluency)	Verbal association fluency (spontaneous word production)
	CTT—2	Time to Completion*	Divided attention, sequencing skills (as well as perceptual tracking, sustained attention, graphomotor skills)
	WCST	Number of Categories Completed	Ability to switch and maintain mental sets, inductive reasoning
		Conceptual Level Responses	Ability to form concept about correct sorting strategy (number of consecutive correct responses of at least 3 or more)
		Total Errors*	Errors in deducing, maintaining and shifting set
		Perseverative Responses*	Measures inability to change set (once matching principle has changed)–responses continued according to previous set
		Perseverative Response Errors*	Measures inability to change set (once matching principle has changed)–responses do not match current set rules
	WMS	Learning Slope I	Verbal memory encoding ability—the increase in recall across consecutive learning trials
**Memory**	BVMT-R	Total Correct Responses	Overall visuospatial recall skills
		Delayed Correct Responses	Long-term visuospatial memory
	CVLT	Total Correct Responses	Global index of verbal learning ability
		Short, and Long, Delay Cued Recall	Short- and long-term ability to recall verbal memories following a given cue
		Short, and Long, Delay Free Recall	Short- and long-term ability to recall verbal memories without a cue
	WMS-III	Logical Memory I (LMI)–Recall (Total Score)	Long-term ability to recall verbal information after one presentation
		Logical Memory II (LMII)–Recall Total Score)	Long-term ability to recall verbal information after two presentations
		LMI 1^st^ Recall Total Score Raw	Short-term ability to recall verbal information after two presentation
		LMII Percent Retention	Measure of long-term verbal memory (information retained)
**Processing Speed**	CTT—1	Time to Completion (Total Seconds)*	Perceptual tracking, sustained attention, graphomotor skills
	Stroop	Colour Score*	Perceptual motor speed
		Word Score*	Perceptual motor speed
**Attention**	PASAT	Correct Responses (Trial 1, 2)	Sustained and divided attention, working memory
**Intellectual Functioning**	WASI	Full Scale Intelligence Quotient	General intelligence

*A higher score indicates a worse performance (e.g. longer time to completion on the CTT– 1 indicates a worse performance than a shorter time to completion)

Both raw scores and standardized t- and/or z-scores were obtained (normative data was obtained from each test’s corresponding administration manual). All analyses was performed using raw data (with the exception of the FSIQ which was age normed). Performance on the Wechsler Abbreviated Scale of Intelligence [55] was compared across groups in order to investigate whether differences in general intelligence quotient (IQ) were statistically significant (and needed to be included as a covariate in data analysis).

### Statistical methods

A double-entry system with independent research personnel was utilized for all cognitive and behavioural data and inconsistencies were checked and resolved by an additional assessor. Any single measure with greater than 30% missing data was excluded from the data set.

Exploratory descriptive group analyses were performed to investigate and characterize group means, ranges, and standard deviations. One-way between group Analysis of Variance (ANOVA) tests were performed on all continuous covariates of interest. Chi-square analyses were run to compare group differences on categorical variables. Significant ANOVA test results were then further investigated by means of pair-wise comparisons (Tukey-HSD).

Although our primary interest was in the effect of obesity on cognition, additional cardiovascular comorbidities may contribute to cognitive performance in obese (bariatric) participants. As such, proportion of comorbidities (e.g., hypertension) was compared between bariatric patient groups to ensure that one group was not heavily loaded with potential confounders. Additionally, comorbidity variables considered potential confounders were explored further in secondary analyses of ANOVA models found to be significant. Pearson product-moment correlation coefficients between cognitive outcomes and medication load composite scores were also calculated.

## Results

### Participants

Participants in the healthy control (HC), bariatric control (BC) and bariatric-MDD (b-MDD) groups did not differ in terms of age, sex distribution, ethnicity or marital status. Although the bariatric groups differed from healthy controls with respect to years of education (F[2,63] = 4.836, p < 0.01), they did not differ on an index measure of intelligence (FSIQ). Critically, when comparing bariatric controls to bariatric MDD participants, no significant group differences were found with regards to the presence of T2D, hypertension, OSA risk, or hyperlipidemia; healthy control participants did not have any of these medical comorbidities. Further exploration indicated that bariatric controls and MDD bariatrics did not differ with regards to weight, BMI, lipid profile levels, blood pressure readings, heart rate, waist and hip circumferences, glucose and HbA1c values. At the time of testing, only one participant from the bariatric control group reported current smoking. With regard to the bariatric MDD group, there were no significant correlations between psychiatric medication load and cognitive performance. See Table 2 for Demographic and medical characteristics of the study sample.

Subjective report of disability impairment, due to obesity and health related problems and/or psychiatric health problems, was significantly different across groups (F[2,61] = 22.24, p < 0.001). In particular, post-hoc Tukey analysis showed that compared to healthy controls, bariatric controls reported an overall level of mild impairment (Mean [M] = 2.65, SD = 2.70, p < 0.001), while bariatric MDD participants reported an overall level of moderate impairment (M = 4.70, SD = 4.81, p < 0.001), on a 10-point scale. Groups also significantly differed on self-reported levels of cognitive impairment on the CFQ (F[2,62] = 12.0 p < 0.001). Pair-wise contrasts showed that the MDD bariatric group reported significantly higher levels of cognitive impairment when compared to both the bariatric control group (p < 0.001) and healthy control group (p < 0.001). Group differences on measures of depression, namely the BDI and HAMD, were also found (F[2,62] = 17.90, p < 0.001 and F[2,62] = 19.12, p < 0.001 respectively). Post-hoc Tukey analysis indicated that in comparison to healthy and bariatric controls, the MDD bariatric group also exhibited significantly higher levels of depression on both self-report (BDI: p < 0.001, p = 0.03, respectively) and clinician-administered scales (HAMD: p < 0.001, p < 0.001, respectively), with the overall group means indicating minimal to mild current depression (BDI: M = 17.5, SD = 10.0; HAMD: M = 6.6, SD = 4.3). Approximately 34.8% of the MDD bariatric group met DSM-IV criteria for current or partially remitted MDD, while the remaining 65.2% were euthymic at the time of testing. BDI and HAMD scores within the MDD bariatric group were not significantly correlated with performance on cognitive test measures. Clinical characteristics of each group are summarized in Table 2.

Participants also did not differ in nutritional intake measures of total daily caloric intake (averaged across 3-days using the FFQ), diet component (diet percentage broken into protein, carbohydrate and fat intake), total fat, cholesterol, total sugar or sodium. Groups did differ in dietary fiber intake (F[2,61] = 3.46, p = 0.04), with healthy controls reporting significantly higher levels (M = 24.0[9.9] grams) as compared to MDD bariatric participants (M = 18.0[5.7]) when examined using post-hoc Tukey analysis but there is no reason to speculate that this alone would impact cognition.

### Neuropsychological performance

No significant group differences were found in intellectual functioning (as assessed by FSIQ score on the WASI) and consequently, FSIQ and education were not added as covariates in cognitive performance ANOVA models. Analyses were conducted using raw data for each measure (with the exception of FSIQ which was standardized according to age normed manual indices). Given the small number of participants who met the minimum PASAT Trial 1 or Trial 2 threshold for administering Trials 3 and 4, group differences were not explored for Trials 3 and 4.

We observed a consistent pattern across measures of memory, executive functioning, attention and processing speed. Specifically, healthy controls consistently performed better than both bariatric groups across the majority of measures. Consistent with our predictions, bariatric controls tended to outperform bariatric MDD patients (see Table 4) across the majority of measures reported. Several of these differences reached statistical significance or showed a trend toward significance (p < 0.10). See Table 3 for description of cognitive variables measured.

**Table 4:** Neuropsychological performance across groups.

Measure Variable	HC (n = 20) Mean (SD)	BC (n = 25) Mean (SD)	b-MDD (n = 21) Mean (SD)
**BVMT–R Total Score** **	29.3 (5.8)	26.6 (5.1)	25.3 (5.8)
**BVMT–R Learning Score** **	3.4 (1.9)	4.3 (1.6)	4.5 (1.6)
**BVMT–R Delayed Score**	11.2 (1.7)	10.4 (1.4)	10.7 (1.4)
**COWAT (FAS) Phonemic**	46.5 (11.9)	40.4 (11.9)	39.3 (12.1)
**COWAT (Animals) Score**	24.3 (4.9)	23.1 (4.6)	22.0 (6.1)
**Color Trails 1 (seconds)**	31.9 (9.5)	33.4 (9.2)	36.9 (11.1)
**Color Trails 2 (seconds)** **	59.3 (17.0)	66.4 (16.6)	74.3 (26.2)
**CVLT-II Total**	49.7 (7.6)	48.8 (8.6)	48.8 (9.2)
**CVLT-II Short-Delay Free Recall**	10.7 (2.6)	10.5 (2.6)	10.0 (2.7)
**CVLT-II Long-Delay Free Recall**	11.5 (2.6)	10.7 (2.5)	10.8 (2.4)
**CVLT-II Short-Delay Cued Recall**	11.7 (2.5)	11.4 (2.0)	11.0 (2.8)
**CVLT-II Long-Delay Cued Recall**	12.1 (2.4)	11.3 (2.4)	11.5 (2.5)
**PASAT Trial 1**	39.7 (13.1)	35.4 (11.9)	32.2 (14.3)
**PASAT Trial 2** **	36.1 (12.5)	29.7 (11.5)	26.8 (11.9)
Stroop Word*	101.4 (11.3)	92.3 (11.6)	89.5 (17.8)
**Stroop Colour** **	75.9 (9.2)	71.4 (10.1)	67.8 (12.8)
Stroop Word-Colour*	45.8 (7.2)	42.0 (8.4)	39.7 (7.7)
**Stroop Interference**	3.7 (6.9)	2.5 (7.9)	1.2 (7.7)
**WCST Total Errors**	14.5 (9.1)	19.8 (11.0)	18.1 (10.0)
**WCST Perseverative Errors**	8.1 (5.4)	11.2 (7.5)	9.3 (4.6)
**WCST Perseverative Responses**	8.9 (6.6)	12.5 (9.4)	10.5 (5.7)
**WCST Conceptual Level Responses**	46.0 (13.3)	38.4 (16.0)	40.3 (15.6)
**WCST Categories Completed**	3.8 (1.9)	2.9 (1.7)	3.1 (1.6)
**WMS-III LMI–Recall**	40.1 (8.4)	39.9 (8.6)	38.6 (11.5)
**WMS-III LMII–Recall**	25.3 (7.7)	24.6 (7.4)	24.4 (8.0)
**WMS-III LMI 1**^**st**^ **Recall Total Score**	24.0 (5.7)	23.4 (6.1)	23.2 (8.0)
WMS-III Learning Slope*	5.2 (2.4)	6.3 (2.1)	4.0 (3.1)
**WMS-III Percent Retention (%)**	84.2 (12.3)	81.5 (13.7)	86.4 (12.2)
**WASI Full Scale IQ**	112.2 (13.6)	107.0 (12.7)	105.3 (12.3)

*significant group effect (p<0.05)

** Trending group effect (p ≤0.10)

All scores presented are raw scores with the exception of “WASI Full Scale IQ” which has been standardized according to age norms.

### Cognitive outcome measures

One-way between group (HC, BC and b-MDD group) ANOVA models revealed group differences across several cognitive measures. Significant group differences emerged on the interference trial of the Stroop Word-Colour Test (F[2,63] = 3.19, p < 0.05). Post-hoc Tukey analysis revealed that the bariatric MDD group showed significant impairment relative to healthy controls on this measure (p = 0.04). Significant group differences were also observed on the Learning Slope index of the WMS-LM1 subtest (F[2,63] = 4.69, p = 0.01). Pair-wise Tukey comparison indicated that bariatric controls outperformed bariatric MDDs (p = 0.01); no other group differences were significant. Group differences in processing speed also emerged on the Stroop word reading subtest (F[2,63] = 4.16, p = 0.02), with a trend towards significance on the Stroop colour identification subtest (F[2,63] = 2.96, p = 0.06). Specifically, the bariatric MDD group showed diminished performance relative to healthy controls (post-hoc Tukey-HSD: p = 0.02 and p = 0.05) on the Stroop Word and Stroop Color subtests, respectively.

In addition, there were trends toward significant group differences on the learning slope and total recall score of the BVMT-R (F[2,63] = 2.65, = 0.08, and F[2,63] = 2.66, p = 0.08, respectively), the Color Trails 2 test (F = [2,62] = 2.81, p = 0.07), and on Trial 2 of the PASAT (F[2,56] = 2.94, p = 0.06). No significant group differences emerged on the CVLT and Color Trails 1.

### Secondary analyses

Given that the MDD bariatric group consisted of currently depressed, partially remitted, and euthymic patients, exploratory analyses was also conducted to assess whether BDI or HAMD scores were significant predicators of cognitive performance outcome measures within the MDD group. BDI and HAMD scores within the MDD bariatric group were not significantly correlated with performance on cognitive test measures.

For measures where a significant difference between the bariatric MDD and healthy control groups was identified, analysis was repeated, splitting the bariatric MDD group into two groups based on the comorbidity effect being investigated (namely, hypertension, T2D or OSA). The same process was completed for the bariatric versus healthy control effect model on the WMS-LM1 Learning Slope score. Hypertension status was found to have a significant effect within the bariatric MDD group only on performance on the Stroop Word measure (F[2,38] = 3.37, p < 0.05). Interestingly however, using post-hoc Tukey analysis, this difference in performance was seen between the bariatric non-hypertensive MDD group compared to healthy controls (p < 0.05), indicating that the presence of hypertension was not responsible for the differences seen between groups in significant ANOVA models. Similarly, the bariatric MDD patients without T2D performed significantly worse than healthy controls on the Stroop Word task (post-hoc Tukey analysis, p = 0.03). Finally, no differences in performance between bariatric MDD patients with and without OSA were found on any of measures examined.

## Discussion

To our knowledge, this is the first study to attempt to examine the potentially deleterious effects of obesity, its comorbidities, and depression on cognitive performance. It is also the first to attempt to control for variables such as medication load, nutritional status and medical illness burden in a systematic way. Provocatively, although healthy controls often outperformed bariatric controls and bariatric controls often outperformed bariatric MDDs (with regards to raw test scores), these differences in cognitive performance did not generally reach significance until comparing performance between healthy controls and bariatric MDDs. This suggests that MDD and obesity may have an additive effect on cognition that leads to measurable deficits in cognitive performance on neuropsychological measures.

Our work adds to the growing number of studies linking obesity to poor cognitive performance [56] and illustrates how even subtle changes in weight can be clinically relevant in populations with preexisting vulnerability. Although obesity may not have a perceptible effect on cognitive performance in psychologically healthy individuals, it may be an issue in patients already susceptible to cognitive dysfunction such as those with MDD.

Consistent with previous studies who have found an effect of obesity on cognition, the majority of our reported findings of cognitive impairment in the bariatric MDD group were on measures of executive functioning, attention, and processing speed, capacities shown to be affected by both obesity and MDD individually [17, 26, 57, 58]. Further, when comparing performance patterns across groups, MDD (including both past and current diagnosis) seemed to have an additive negative effect on cognition in the presence of obesity, with comparisons between bariatric MDD and healthy controls resulting in significantly different levels of performance on several cognitive tasks (differences were generally not seen as significant when contrasting bariatric MDD and bariatric controls). However, it is possible that subtle differences in group performances that do not meet the threshold for statistical significance may still result in clinically significant impact. This has important ramifications on both clinical practice and theoretical research.

Clinically, many psychotropic medications are associated with problematic metabolic side effects, including increased weight gain [59, 60]. A common complaint of MDD patients is cognitive impairment [15]; in line with this, the MDD sample in our study reported high levels of subjective cognitive impairment on the CFQ. Current clinical treatment guidelines do not factor in tolerability when suggesting first and second line options, but knowing metabolic changes can impact cognitive functioning (an outcome used as a measure of remission of psychiatric symptomatology) may impact stratification of medication recommendations, especially given the emphasis on functional recovery [27]. Given an option between several first-line treatment options for MDD, physicians may want to therefore consider a psychotropic medication with a smaller likelihood of significant weight gain, as well as implementing a weight management or monitoring strategy with medication-treated patients.

While it is known that MDD is associated with impairment on tests of memory, attention, executive function and processing speed [11, 12, 26] few studies investigating interactions between MDD and cognitive impairment report on, or account for, the possible confounding effects of obesity. In our study, we found that an additive effect of MDD and obesity reached significance on measures of processing speed and executive function. One can therefore speculate that some of the cognitive impairment traditionally attributed to the presence of a mood disorder may be associated with obesity and/or its related metabolic comorbidities and further work is needed to articulate this linkage.

## Limitations

Although a consistent group performance pattern was seen across almost all measures, results may have failed to achieve significance on certain measures due to our study’s modest sample size limitation. Also, due to the absence of a non-bariatric MDD study group, we are unable to draw complete conclusions regarding the independent effects (and interactions) between MDD and obesity on cognition. However, our exploratory yet compelling results (while controlling for extraneous variables such as medication load, nutritional status and common metabolic comordidities) strongly urge for further investigation and study replication with larger sample sizes (along with the addition of a non-bariatric MDD group).

## Conclusion

We observed an overall consistent pattern across several neuropsychological wherein healthy controls consistently achieved higher scores than both bariatric groups (BC and MDD-B) across the majority of test measures, while the BC group tended to outperform the MDD-B group. This indicates that obesity may partially contribute to the negative impairment associated with MDD. Further work is needed to expand on these provocative findings to clarify the directionality between obesity and cognitive impairment and to further disentangle the interactive effects of mood disorder diagnosis, obesity and cognitive performance. The work to date, however, indicates that obesity may impact cognitive performance in individuals with preexisting vulnerability, such as those with MDD, and weight management is especially important in this population in order to ensure the best outcomes.

## References

[1] Costa E, Santos-Silva A, Paul C, Gonzalez Gallego J. Aging and cardiovascular risk. BioMed Research International. 2015;2015(871656). 10.1155/2015/871656. PMC4363687 25821825

[2] Janssen I. The public health burden of obesity in Canada. Canadian Journal of Diabetes. 2013;37(2):90–6. 10.1016/j.jcjd.2013.02.059 24070798

[3] Ferrari AJ, Charlson FJ, Norman RE, Patten SB., Freedman G, Murray CJL, et al Burden of Depressive Disorders by Country, Sex, Age, and Year: Findings from the Global Burden of Disease Study 2010. PLoS Medicine. 2013;10(11):e1001547 Epub November 5, 2013. 10.1371/journal.pmed.1001547 24223526 PMC3818162

[4] Simon GE, Von Korff M, Saunders K, Miglioretti DL, Crane PK, van Belle G, et al Association Between Obesity and Psychiatric Disorders in the US Adult Population. Archives of General Psychiatry 7 2006;63(7):824–30. 10.1001/archpsyc.63.7.824 16818872 PMC1913935

[5] Alonso-Alonso M, Pascual-Leone A. The right brain hypothesis for obesity. Journal of the American Medical Association. 2007;297(16):1819–22. 10.1001/jama.297.16.1819. 17456824

[6] Alosco ML, Galioto R, Spitznagel MB, Strain G, Devlin M, Cohen R, et al Cognitive function after bariatric surgery: Evidence for improvement 3 years after surgery. American Journal of Surgery. 2014;207(6):870–6. 10.1016/j.amjsurg.2013.05.018. 24119892 PMC3983172

[7] Benito-Leon J, Mitchell AJ, Hernandez-Gallego J, Bermejo-Pareja F. Obesity and impaired cognitive functioning in the elderly: A population-based cross-sectional study (NEDICES). European Journal of Neurology. 2013;20(6):899–906. 10.1111/ene.12083. 23323838

[8] Beth Spitznagel M, Alosco M, Strain G, Devlin M, Cohen R, Paul R, et al Cognitive function predicts 24-month weight loss success after bariatric surgery. Surgery for Obesity and Related Diseases. 2013;9(5):765–70. 10.1016/j.soard.2013.04.011. 23816443 PMC3788845

[9] Boeka AG, Lokken KL. Neuropsychological performance of a clinical sample of extremely obese individuals. Archives of Clinical Neuropsychology. 2008;23(4):467–74. 10.1016/j.acn.2008.03.003. 18448310

[10] Smith E, Hay P, Campbell L, Trollor JN. A review of the association between obesity and cognitive function across the lifespan: Implications for novel approaches to prevention and treatment. Obesity Reviews. 2011;12(9):740–55. 10.1111/j.1467-789X.2011.00920.x. 21991597

[11] Rock P, Roiser J, Riedel W, Blackwell A. Cognitive impairment in depression: a systematic review and meta-analysis. Psychological medicine. 2014;44(10):2029–40. 10.1017/S0033291713002535 24168753

[12] Trivedi MH, Greer TL. Cognitive dysfunction in unipolar depression: Implications for treatment. Journal of Affective Disorders.152–154(Complete):19–27. 10.1016/j.jad.2013.09.012 24215896

[13] Snyder HR. Major depressive disorder is associated with broad impairments on neuropsychological measures of executive function: a meta-analysis and review. Psychological bulletin. 2013;139(1):81 10.1037/a0028727 22642228 PMC3436964

[14] Snyder HR, Miyake A, Hankin BL. Advancing understanding of executive function impairments and psychopathology: bridging the gap between clinical and cognitive approaches. Frontiers in psychology. 2015;6. 10.3389/fpsyg.2015.00328 PMC4374537 25859234

[15] McDermott LM, Ebmeier KP. A meta-analysis of depression severity and cognitive function. Journal of affective disorders. 2009;119(1):1–8. 19428120 10.1016/j.jad.2009.04.022

[16] Talarowska M, Florkowski A, Zboralski K, Berent D, Wierzbiński P, Gałecki P. Auditory-verbal declarative and operating memory among patients suffering from depressive disorders–preliminary study. Advances in medical sciences. 2010;55(2):317–27. 10.2478/v10039-010-0053-0 21163755

[17] Harvey PO, Le Bastard G, Pochon JB, Levy R, Allilaire JF, Dubois B, et al Executive functions and updating of the contents of working memory in unipolar depression. J Psychiatr Res. 2004;38(6):567–76. 10.1016/j.jpsychires.2004.03.003 15458852

[18] Schoedel KA, Addy C, Chakraborty B, Rosko K, Dunbar S, Maes A, et al Human abuse potential and cognitive effects of taranabant, a cannabinoid 1 receptor inverse agonist: A randomized, double-blind, placebo-and active-controlled, crossover study in recreational polydrug users. Journal of Clinical Psychopharmacology. 2012;32(4):492–502. 10.1097/JCP.0b013e31825d380d. 22722508

[19] Simon GE, Ludman EJ, Linde JA, Operskalski BH, Ichikawa L, Rohde P, et al ASSOCIATION BETWEEN OBESITY AND DEPRESSION IN MIDDLE-AGED WOMEN. General hospital psychiatry. 2008;30(1):32–9. 10.1016/j.genhosppsych.2007.09.001 18164938 PMC2675189

[20] McIntyre RS, Konarski JZ, Wilkins K, Soczynska JK, Kennedy SH. Obesity in bipolar disorder and major depressive disorder: results from a national community health survey on mental health and well-being. Canadian journal of psychiatry. 2006;51(5):274 10.1177/070674370605100502 16986816

[21] Restivo MR, McKinnon MC, Frey BN, Hall G, Taylor V. Cognitive profile of obese adults with and without a mood disorder. Biological Psychiatry. 2014;1):55S 10.1016/j.biopsych.2014.03.014.

[22] Hasselbalch BJ, Knorr U, Kessing LV. Cognitive impairment in the remitted state of unipolar depressive disorder: a systematic review. Journal of affective disorders. 2011;134(1):20–31. 21163534 10.1016/j.jad.2010.11.011

[23] Lee RS, Hermens DF, Porter MA, Redoblado-Hodge MA. A meta-analysis of cognitive deficits in first-episode major depressive disorder. Journal of affective disorders. 2012;140(2):113–24. 10.1016/j.jad.2011.10.023 22088608

[24] McLennan SN, Mathias JL. The depression-executive dysfunction (DED) syndrome and response to antidepressants: a meta-analytic review. International journal of geriatric psychiatry. 2010;25(10):933–44. 10.1002/gps.2431 20872927

[25] Wagner S, Doering B, Helmreich I, Lieb K, Tadić A. A meta-analysis of executive dysfunctions in unipolar major depressive disorder without psychotic symptoms and their changes during antidepressant treatment. Acta Psychiatrica Scandinavica. 2012;125(4):281–92. 10.1111/j.1600-0447.2011.01762.x 22007857

[26] Bora E, Harrison BJ, Yücel M, Pantelis C. Cognitive impairment in euthymic major depressive disorder: a meta-analysis. Psychological Medicine. 2013;43(10):2017–26. 10.1017/S0033291712002085 23098294

[27] McIntyre RS, Liauw S, Taylor VH. Depression in the workforce: The intermediary effect of medical comorbidity. Journal of Affective Disorders. 2011;128(SUPPL. 1):S29–S36. 10.1016/S0165-0327(11)70006-4. 21220078

[28] Oliveira de Lima Queiroz L, Junqueira AX, Fontana AM, De Oliveira ER, Lima VC, Guarienti VC. Prevention of cognitive impairment through a cognitive stimulation and rehabilitation program mediated by computers and internet. Journal of the Neurological Sciences. 2013;333:e537 10.1016/j.jns.2013.07.1889.

[29] Restivo MR, McKinnon MC, Frey BN, Hall GB, Taylor VH. Effect of obesity on cognition in adults with and without a mood disorder: study design and methods. BMJ Open. 2016;6(2). 10.1136/bmjopen-2015-009347 PMC4780057 26928024

[30] WHO. WHO STEPwise approach to surveillance (STEPS). Geneva, Switzerland: World Health Organization (WHO); 2008.

[31] Anvari M SA, Yusef S. Registry data produced and distributed by the Population Health Research Institute and the Centre for Surgical Invention and Innovation, supported by the Ministry of Health and Long-Term Care. 2015.

[32] Pi-Sunyer FX. NHLBI Obesity Education Initiative Expert Panel on the identification, evaluation, and treatment of overweight and obesity in adults-The evidence report. Obesity Research. 1998;6:51S–209S. 9813653

[33] First MB, Spitzer RL, Gibbon M, Williams JB. SCID-I/P. 2007.

[34] Chung F, Yegneswaran B, Liao P, Chung SA, Vairavanathan S, Islam S, et al Validation of the Berlin questionnaire and American Society of Anesthesiologists checklist as screening tools for obstructive sleep apnea in surgical patients. The Journal of the American Society of Anesthesiologists. 2008;108(5):822–30–30. 10.1097/ALN.0b013e31816d91b5 18431117

[35] Willett WC, Leibel RL. Dietary fat is not a major determinant of body fat. The American Journal of Medicine. 2002;113(9):S47–59. 10.1016/s0002-9343(01)00992-5 12566139

[36] Sackeim HA. The definition and meaning of treatment-resistant depression. Journal of Clinical Psychiatry. 2001;62:10–7. 11480879

[37] Hassel S, Almeida JR, Kerr N, Nau S, Ladouceur CD, Fissell K, et al Elevated striatal and decreased dorsolateral prefrontal cortical activity in response to emotional stimuli in euthymic bipolar disorder: no associations with psychotropic medication load. Bipolar disorders. 2008;10(8):916–27. 10.1111/j.1399-5618.2008.00641.x 19594507 PMC2711546

[38] Broadbent DE, Cooper PF, FitzGerald P, Parkes KR. The cognitive failures questionnaire (CFQ) and its correlates. British Journal of Clinical Psychology. 1982;21(1):1–16. 7126941 10.1111/j.2044-8260.1982.tb01421.x

[39] Sheehan DV, Harnett-Sheehan K, Raj BA. The measurement of disability. International Clinical Psychopharmacology June. 1996;3:89–95. 10.1097/00004850-199606003-00015 8923116

[40] Beck AT, Steer RA, Brown GK. Manual for Beck Depression Inventory II (BDI II). San Antonio, Texas: Psychology Corporation; 1996.

[41] Hedlung JL, Vieweg BW. The Hamilton rating scale for depression: A comprehensive review. Journal of Operational Psychiatry. 1979;10(2):149–65.

[42] Altman EG, Hedeker D, Peterson JL, Davis JM. The Altman self-rating mania scale. Society of Biological Psychiatry 1997;42:948–55. 10.1016/S0006-3223(96)00548-3 9359982

[43] Young RC, Biggs JT, Ziegler VE, Meyer DA. A rating scale for mania: Reliability, validity and sensitivity. British Journal of Psychiatry. 1978;133(11):429–35. 728692 10.1192/bjp.133.5.429

[44] Berstein DP, Fink L. Childhood Trauma Questionnaire: A retrospective self-report manual. San Antonio, Texas: Harcourt Brace & Company; 1998.

[45] Elias MF, Elias PK, Sullivan LM, Wolf PA, D'Agostino RB. Lower cognitive function in the presence of obesity and hypertension: The Framingham heart study. International Journal of Obesity. 2003;27(2):260–8. 10.1038/sj.ijo.802225. 12587008

[46] van den Berg E, Kloppenborg RP, Kessels RP, Kappelle LJ, Biessels GJ. Type 2 diabetes mellitus, hypertension, dyslipidemia and obesity: A systematic comparison of their impact on cognition. Biochimica et biophysica acta. 2009;1792(5):470–81. 10.1016/j.bbadis.2008.09.004 18848880

[47] Delis DC, Kramer JH, Kaplan E, Ober BA. California Verbal Learning Test: Adult Version. San Antonio, Texas: Harcourt Brace & Company; 1987.

[48] Wechsler D. Wechsler Memory Scale Third Edition ed. San Antonio, TX: The Psychological Association; 1997.

[49] Benedict RHB. Brief Visuospatial Memory Test—Revised: Manual. Odessa, Florida: Psychological Assessment Resources; 1997.

[50] Benton AL, Hamsher K, Sivan AB. Multilingual Aphasia Examination. 3rd ed Iowa City, Iowa: AJA Associates; 1983.

[51] Golden JC. Stroop Color and Word Test. Chicago, Illinois: Stoelting Company; 1978.

[52] Heaton EK, Chelune GJ, Talley JL, Kay GG, Curtis G. Wisconsin Card Sorting Test (WCST) Manual Revised and Expanded. Odessa, Florida: Psychological Assessment Resources; 1993.

[53] Reitan RM, Wolfson D. The Halstead-Reitan Neuropsychological Test Battery. Tucson, AZ: Neuropsychology Press; 1985.

[54] Gronwall D. Paced Auditory Serial-Addition Task: A measure of recovery form concussion. Perceptual and Motor Skills. 1977;44:367–73. 10.2466/pms.1977.44.2.367 866038

[55] Wechsler D. Wechsler Abbreviated Scale of Intelligence (WASI) San Antonio, TX: NCS Pearson Inc.; 1999.

[56] Liang J, Matheson BE, Kaye WH, Boutelle KN. Neurocognitive correlates of obesity and obesity-related behaviors in children and adolescents. International Journal of Obesity. 2014;38(4):494–506. 10.1038/ijo.2013.142. 23913029 PMC4456183

[57] Cook R, O'Dwyer N, Steinbeck K, Rooney K, O'Connor H. Obesity, cognitive functioning and mental health in young women. Obesity Facts. 2013;6:173.

[58] Jean MDK, Ajilore O. Executive dysfunction in type 2 diabetes: The role of high blood pressure and body mass index. Diabetes. 2014;63:A212 10.2337/db14-665-832.

[59] Barrett-Connor E. The association between depression and diabetes. Cardiology (Switzerland). 2013;125:94 10.1159/000354059.

[60] Birt J. Management of weight gain associated with antipsychotics. Annals of Clinical Psychiatry. 2003;15(1):49–58. 10.1023/A:1023280610379. 12839432

